# Comparative Study of Salivary, Duodenal, and Fecal Microbiota Composition Across Adult Celiac Disease

**DOI:** 10.3390/jcm9041109

**Published:** 2020-04-13

**Authors:** Simona Panelli, Enrica Capelli, Giuseppe Francesco Damiano Lupo, Annalisa Schiepatti, Elena Betti, Elisabetta Sauta, Simone Marini, Riccardo Bellazzi, Alessandro Vanoli, Annamaria Pasi, Rosalia Cacciatore, Sara Bacchi, Barbara Balestra, Ornella Pastoris, Luca Frulloni, Gino Roberto Corazza, Federico Biagi, Rachele Ciccocioppo

**Affiliations:** 1 Department of Biomedical and Clinical Sciences “L. Sacco”, Pediatric Clinical Research Center “Invernizzi”, University of Milan, 20122 Milan, Italy; simona.panelli1@unimi.it; 2 Laboratory of Immunology and Genetic Analysis, Department of Earth and Environmental Science, University of Pavia, 27100 Pavia, Italy; enrica.capelli@unipv.it (E.C.); giuseppe.lupo01@universitadipavia.it (G.F.D.L.); sara.bacchi01@universitadipavia.it (S.B.); 3 Centre for Health Technologies, University of Pavia, 27100 Pavia, Italy; simone.marini@unipv.it (S.M.); riccardo.bellazzi@unipv.it (R.B.); 4 Department for Sustainable Food Processes, Università Cattolica del Sacro Cuore, 29122 Piacenza, Italy; 5 Gastroenterology Unit, I.R.C.C.S. Pavia, I.C.S. Maugeri, University of Pavia, 27100 Pavia, Italy; salinana@hotmail.it (A.S.); federico.biagi@icsmaugeri.it (F.B.); 6 Department of Internal Medicine and Therapeutics, Fondazione I.R.C.C.S. Policlinico San Matteo and University of Pavia, 27100 Pavia, Italy; elena.betti19@gmail.com (E.B.); gr.corazza@smatteo.pv.it (G.R.C.); 7 Laboratory of Bioinformatics, Mathematical Modelling and Synthetic Biology, Department of Electrical, Computer and Biomedical Engineering, University of Pavia, 27100 Pavia, Italy; elisabetta.sauta01@universitadipavia.it; 8 Unit of Anatomic Pathology, Department of Molecular Medicine, University of Pavia and Fondazione I.R.C.C.S. Policlinico San Matteo, 27100 Pavia, Italy; a.vanoli@smatteo.pv.it; 9 Laboratory of Immunogenetics, Department of Transfusion Medicine and Immuno-Hematology, Fondazione I.R.C.C.S. Policlinico S. Matteo, 27100 Pavia, Italy; a.pasi@smatteo.pv.it (A.P.); r.cacciatore@smatteo.pv.it (R.C.); 10 Laboratory of Pharmacogenetics, Department of Biology and Biotechnology, University of Pavia, 27100 Pavia, Italy; barbara.balestra@unipv.it (B.B.); ornella.pastoris@unipv.it (O.P.); 11 Gastroenterology Unit, Department of Medicine, A.O.U.I. Borgo Roma and University of Verona, 37134 Verona, Italy; luca.frulloni@univr.it

**Keywords:** celiac disease, enteropathy, microbiota, gluten, therapy

## Abstract

Background: Growing evidence suggests that an altered microbiota composition contributes to the pathogenesis and clinical features in celiac disease (CD). We performed a comparative analysis of the gut microbiota in adulthood CD to evaluate whether: (i) dysbiosis anticipates mucosal lesions, (ii) gluten-free diet restores eubiosis, (iii) refractory CD has a peculiar microbial signature, and (iv) salivary and fecal communities overlap the mucosal one. Methods: This is a cross-sectional study where a total of 52 CD patients, including 13 active CD, 29 treated CD, 4 refractory CD, and 6 potential CD, were enrolled in a tertiary center together with 31 controls. A 16S rRNA-based amplicon metagenomics approach was applied to determine the microbiota structure and composition of salivary, duodenal mucosa, and stool samples, followed by appropriate bioinformatic analyses. Results: A reduction of both α- and β-diversity in CD, already evident in the potential form and achieving nadir in refractory CD, was evident. Taxonomically, mucosa displayed a significant abundance of *Proteobacteria* and an expansion of *Neisseria*, especially in active patients, while treated celiacs showed an intermediate profile between active disease and controls. The saliva community mirrored the mucosal one better than stool. Conclusion: Expansion of pathobiontic species anticipates villous atrophy and achieves the maximal divergence from controls in refractory CD. Gluten-free diet results in incomplete recovery. The overlapping results between mucosal and salivary samples indicate the use of saliva as a diagnostic fluid.

## 1. Introduction

The discovery of the gut microbiota universe and the growing understanding of its role in health and disease have radically changed the current point of view on the pathogenesis of noncommunicable diseases [1]. Among these, celiac disease (CD) represents a privileged situation since both the external (gluten) and internal (tissue transglutaminase) antigens as well as the predisposing human leukocyte antigen haplotypes have been identified [2]. However, although gluten is widely ingested, tissue transglutaminase is a ubiquitous enzyme, and the frequency of at-risk alleles in the general population approaches 40%, only a small proportion of subjects eventually develop enteropathy [3]. Additional factors have therefore been invoked to explain the onset and maintenance of loss of gluten tolerance and mucosal damage. Recently, evidence has been accumulated on the presence of perturbations of the microbiota composition [4] not only in active CD (ACD) [5] but also in a consistent proportion of treated patients (TCD) [6]. Whether dysbiosis represents an epiphenomenon of the enteropathy or, conversely, it contributes to the development of mucosal damage still remains unknown. 

On this basis, we firstly aimed to characterize the mucosal microbiota of an adult CD population, including ACD, TCD, refractory CD (RCD), and potential CD (PCD), and compare it to non-CD controls by using the amplicon metagenomics approach. Secondly, since it is unlikely that the most studied bacterial consortium, i.e., fecal microbiota, represents the composition at the level of duodenal mucosa, we also collected and analysed salivary samples to assess whether they mirror the profile at mucosal level better than feces.

## 2. Patients and Methods

### 2.1. Study Population

A total of 83 cases were recruited at the Department of Internal Medicine, Fondazione I.R.C.C.S. Policlinico San Matteo (Pavia, Italy) from November 1st, 2015 to June 30th, 2018. They included 52 CD patients, comprehensive of all the possible combinations of diet and villous atrophy (Table 1), i.e., 13 ACD, 29 TCD, 4 RCD, 6 PCD, and 31 patients with functional dyspepsia [7] who served as controls (C). The demographic and clinical features of all recruited cases are shown in Table 2. No significant differences regarding age and comorbidities were found among the groups, albeit the statistical analysis was not performed with the potential and refractory ones due to the small sample size. Diagnoses of PCD, ACD, TCD, and RCD were made on the basis of widely accepted criteria [8] and histological examination of mucosal lesions [9]. The adherence to gluten-free diet (GFD) was evaluated by means of a five-level score, with scores ranging 0 to 2 meaning absence or poor adherence and scores ranging 3 to 4 being indicative of good adherence [10]. Moreover, all enrolled cases followed a Mediterranean diet, and there were no vegan/vegetarian patients.

Patients were excluded if they had recent (within 4 weeks) or current use of medications that could affect bowel function and/or microbiota composition, such as antibiotics, prebiotics, probiotics, opioids, nonsteroidal anti-inflammatory drugs, proton pump inhibitors, laxatives, steroids, or antidiarrheal drugs. 

The protocol was approved by the local Ethics Committee (protocol number 20150003822), and each enrolled case gave written informed consent.

### 2.2. Biological Samples 

Saliva

Saliva samples were collected by direct spitting into a sterile plastic tube at least two hours after tooth brushing and before endoscopy. Smoking, and food and drink intake were forbidden since midnight. After collection, salivary samples were kept at −80 °C till analysis.

Mucosa

In all cases, four perendoscopic specimens from duodenal mucosa were formalin-fixed and paraffin-embedded for traditional histology and immunohistochemistry, while two samples were snap-frozen and stored at −80 °C until use. In RCD patients, four additional biopsies were collected for intraepithelial lymphocyte phenotyping.

Stool

Samples were collected at home by patients within 24 h before the endoscopy and kept at −20 °C till delivery in the hospital where they were frozen at −80 °C.

### 2.3. Extraction and Quantification of DNA

DNA was extracted from each biological sample by using commercial kits (all from Qiagen; Hilden, Germany) and following the manufacturer’s instructions according to the suggested procedure for bacteria (DNeasy^®^ Blood & Tissue Handbook). Specifically, the QIAamp DNA Stool Mini kit was used for feces; the QIAamp DNA Blood Mini Kit was used for saliva; and the DNeasy Blood and Tissue Kit was used for duodenal biopsies. More in depth, stool samples were first solubilized in a buffer provided in the kit in order to remove polymerase chain reaction inhibitors contained in feces; then, the DNA extraction was performed applying the same procedures used for the other sample biotypes. The DNA concentration of each sample was assessed fluorometrically.

### 2.4. Production of 16S rRNA Amplicons (V3–V4 Regions) and Sequencing

For amplicon production, the V3–V4 hypervariable regions of the prokariotic 16S rRNA gene were targeted [11]. Polymerase chain reaction was performed in a 50-μL volume containing template DNA, 1× HiFi HotStart Ready Mix (Kapa Biosystems; Wilmington, MA, USA) and 0.5 μM of each primer. The cycling program, performed on a MJ Mini thermal cycler (Promega corp.; Madison, WI), included an initial denaturation cycle (95 °C for 3 min), followed by a variable number of cycles (25 for saliva; 30 for feces and mucosa) at 94 °C for 30 sec, at 55 °C for 30 sec, at 72 °C for 30 sec, and at a final extension (72 °C for 5 min) [12,13]. Cleanup of amplicons was performed using Agencourt AMPure XP SPRI magnetic beads (ThermoFisher Scientific; Waltham, MA, USA). Illumina sequencing libraries were finally constructed through the link of indices (Nextera XT Index Kit, Illumina; San Diego, CA, USA), quantified using a Qubit 2.0 Fluorometer (ThermoFisher Scientific), normalized, and pooled. Libraries underwent paired-end sequencing on an Illumina MiSeq platform at BMR Genomics (Padua, Italy).

### 2.5. Power Calculation

In this cross-sectional observational study, a convenience sample of 80 cases will be enrolled, possibly distributed as follows: 10 potential CD patients, 10 complicated CD patients, 20 active CD patients, 20 treated CD patients, and 20 non-CD patients. The effect size (mean difference/standard deviation) that can be elicited, given the sample size and 80% power, was computed based on the primary endpoint, i.e., the comparison of the microbiota composition between CD groups (potential, active, treated, and refractory) and non-CD patients (controls). A conservative alpha of 0.001 was used, given the multiple endpoints and comparisons planned. The effect size that can be discovered on the basis of these hypotheses will be 1.8 when comparing potential and treated patients to controls and 1.4 when comparing active and treated patients to controls.

### 2.6. Bioinformatics Analysis

An ad hoc bioinformatics pipeline was built up under the R environment [14] Raw sequences were processed using USEARCH (version 10.0.240). Paired reads were merged, and low-quality reads were discarded. Filtered reads were assigned to different taxonomic levels (from phylum to species) and organised into operational taxonomic units (OTUs). Sequences were clustered at 97% nucleotide similarity, and chimeric ones were filtered out; their taxonomy was assessed through the Greengenes 16S rRNA bacterial database (version 13.8) [15]. Data were normalized with the Total Sum Scaling method, and normalized OTUs were used to investigate community diversity in each sample biotype. The observed richness and the Chao1 [16] and Shannon [17] indices were calculated to analyse the within-sample species richness (α-diversity). The β-diversity analysis was conducted to estimate the between-sample diversity, using the generalized UniFrac index as a distance metric [18]. The resulting phylogenetic matrices were represented by multidimensional scaling. Permutational Multivariate Analysis of Variance (PERMANOVA) was performed for β-diversity analysis to statistically assess the grouping of samples by diagnosis. Microbial profiles obtained for each taxonomic level and for each sample biotype were compared among patient groups using the Mann–Whitney U-test, the Kruskal–Wallis Rank Sum test, and a 20% cutoff for prevalence. For all the statistical analyses, the significance threshold (*p*-value) was set to 0.05, and all the obtained *p* values were corrected for multiple testing with the Benjamini–Hochberg method.

## 3. Results

### 3.1. Overall Structure of Salivary, Mucosal, and Fecal Bacterial Communities 

**Figure 1:**
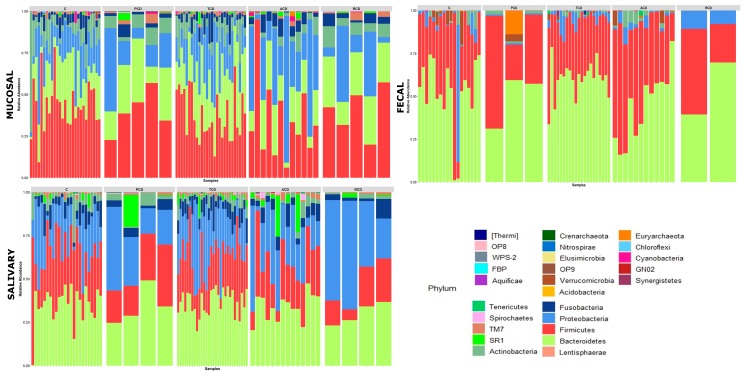
Phylum-level classification of bacteria identified in individual mucosal, salivary, and fecal samples belonging to the five study groups: Each bar represents the relative contribution of phylum-level profiles of each subject enrolled in the study as indicated on the top of each panel (ACD: active celiac disease, C: control subjects, PCD: potential celiac disease, RCD: refractory celiac disease, and TCD: treated celiac disease). Twenty-six different phyla were identified across the three biotypes and are represented by different colors as indicated in the legend. The relative distribution of phyla among study groups indicates a critical decrease of *Firmicutes* and *Actinobacteria* and an expansion of *Proteobacteria* at mucosal and salivary levels in all celiac groups, mostly in ACD and RCD, in comparison with the C group that does not normalize in TCD. By contrast, no clear differences are evident in the stools.

**Figure 2:**
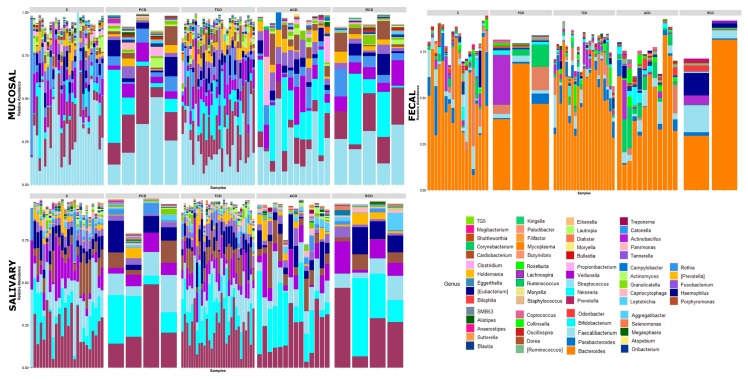
Genus-level classification of bacteria identified in individual mucosal, salivary, and fecal samples belonging to the five study groups: Each bar represents the relative contribution of genus-level profiles with an abundance of at least 1% in each considered sample as indicated on the top of each panel. Sixty-nine different genera were identified across the three biotypes and are represented by different colors as indicated in the legend. An expansion of *Neisseria* and a reduction of *Streptococcus* in celiac groups with respect to controls emerge in both mucosal and salivary communities, whereas *Bacteroides* is the predominant one in the stool consortium.

To investigate the shift in structure and composition of the mucosal, salivary, and fecal bacterial communities across patient groups, 209 out of 249 expected samples (77 mucosal, 76 salivary, and 56 fecal) underwent sequencing and processing, since in some cases (one ACD, two C, and three TCD) the samples displayed degradation of the nucleic acid due to poor conservation. A total of 3.5 million high-quality reads were obtained, of which 5,327,971 were for mucosal, 4,993,425 were for salivary, and 3,208,768 were for fecal samples. All the obtained sequences were classified into 5556 OTUs, representing 26 phyla, 52 classes, 89 orders, 156 familiae, 315 genera, and 186 species (spp.) (see Table S1: Taxonomic assignment across sample biotypes in the study cohort). The relative distribution of the phyla is shown in Figure 1, where a critical decrease of *Firmicutes* and *Actinobacteria* and an expansion of *Proteobacteria* at mucosal and salivary levels appear evident in all CD groups, mostly in ACD and RCD, in comparison with the C group that does not normalize in TCD. By contrast, no clear differences are evident at the stool level probably due to the high variability among samples. As regards the relative distribution of genera, as in Figure 2, an expansion of *Neisseria* and a reduction of *Streptococcus* in CD groups with respect to the C group emerge in both mucosal and salivary communities, whereas *Bacteroides* is the predominant one in the stool consortium.

**Figure 3:**
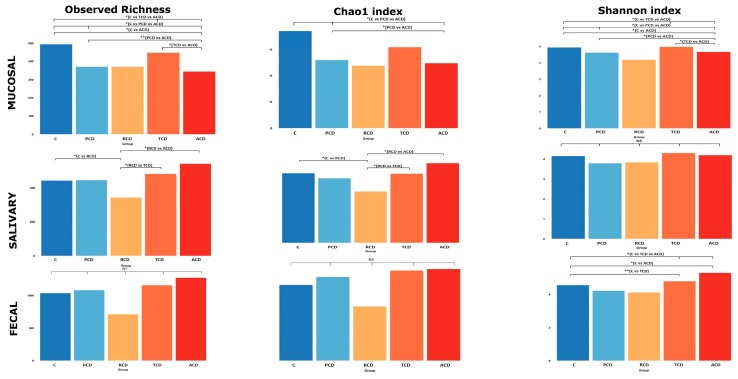
Alpha-diversity: Overall comparison of mucosal, salivary, and fecal microbiota structure. Observed richness and the Chao1 (representing community richness) and Shannon (representing diversity) indices are presented. The bars depict the mean of relative abundances rates. Significant (* *p* < 0.05; ** *p* < 0.01) comparisons between patient categories are indicated over the bars.

The within-sample diversity (α-diversity) was evaluated by computing observed richness and the Chao1 and Shannon indices, of which the single values are shown in Table S2: α-diversity indices for each sample. Figure 3 shows a critical reduction of both observed richness and Shannon index in all CD groups in comparison to the C group at the mucosal level, while the Chao1 index yields significant differences when comparing ACD and PCD with C. The group that fails to be significantly discriminated despite the apparent reduction is the RCD one, possibly because of the small sample size. The salivary community shows the highest richness and Chao1 index in ACD and the lowest in RCD, while PCD and TCD display values similar to the C group. At variance with salivary samples at the fecal level, only the Shannon index produces significant differences between groups, with ACD and TCD showing higher values than C. 

**Figure 4:**
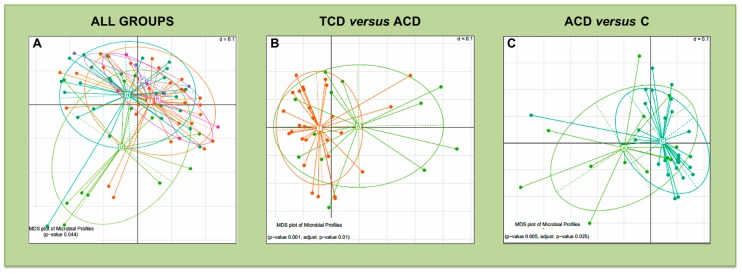
Beta-diversity comparison for the mucosal microbiota: Multidimensional Scaling (MDS) plots of mucosal samples for which the Permutational Multivariate Analysis of Variance test detected a significant separation of study groups in terms of bacterial community composition. The microbiota phylogenetic distances were evaluated through the generalized UniFrac distance. Each point represents the microbiota composition of one sample. Panel (**A**): all patient groups were compared. Panel (**B**): TCD versus ACD comparison. Panel (**C**): ACD versus C comparison. Each point represents the microbiota composition of one sample.

The comparison of β-diversity reveals significant differences only in mucosal bacterial community distribution, when considering all groups of patients (Figure 4A) and the following pairwise comparisons: TCD versus ACD (Figure 4B), and ACD versus C (Figure 4C). 

### 3.2. Taxonomy-Based Analyses of Mucosal Community

As shown in Table 3, the phylum distribution is indicative of a decrease in *Actinobacteria* and an increase in *Proteobacteria* in all celiac groups, with ACD being that with the greatest deviation with respect to C. A different pattern is observed for *Bacteroidetes* that are decreased in ACD and increased in the other CD groups in comparison to C. Although *Firmicutes* are the most abundant phylum, accounting for a portion of bacterial diversity ranging from a maximum of 43.6% in C to a minimum of 34.8% in ACD, with the other CD groups displaying intermediate values (39.5% in PCD, 37.5% in TCD, and 40.16% in RCD), no significant differences are seen. Moving on to bacterial families within the last phylum, the *Streptococcaceae* show the lowest value in TCD, the *Veillonellaceae* are the lowest in ACD, and the *Lachnospiraceace* are the lowest in both ACD and RCD, whereas a critical decrease of *Gemellaceae* in PCD, ACD, and TCD in comparison to C and RCD is clearly evident. Within *Bacteroidetes*, only the *Prevotellaceae* show differences, with ACD displaying a decrease and the other CD groups displaying an increase in comparison to C. Concerning *Actinobacteria*, the *Micrococcaceae* show a critical reduction in CD groups, achieving the nadir in ACD, whilst RCD mirrors C. Finally, within the *Proteobacteria*, *Neisseriaceae* show a robust increase in all CD groups, mostly in ACD, in comparison with C. This situation is perfectly overlapped by the profile observed for the genus *Neisseria*. Among the other genera, *Streptococcus* spp. presents an opposite trend, since it is reduced in CD, especially in ACD and, to a lesser extent, in TCD, while the relative abundance found in PCD and RCD was closer to the profile detected in C. Within the same phylum of *Firmicutes* (*Peptoniphilaceae* family), *Parvimonas* spp. shows a particular profile, with a value in PCD double the level found in C, while it is the half of the C group in the other CD groups. At the species level, it is interesting to note that *Prevotellae* increase in all CD groups with respect to C, except in ACD. By contrast, *Rothia aerea* displays values in CD groups similar to C, except in PCD, where its relative abundance is decreased, and in RCD, where it appears increased.

### 3.3. Taxonomy-Based Analyses of Salivary Community

Also, in this ecosystem, *Firmicutes* is the most represented phylum, accounting for 33% of the bacterial diversity in C and falling to around 25% in TCD, ACD, and PCD and to 17% in RCD. *Bacteroidetes*, on the other hand, show more homogeneous values, ranging from 30.04% in RCD to 36.6% in TCD, with the other groups being around 33%. Interestingly, the *SR1* phylum displays a relative abundance <1% in both C (0.96%) and RCD (0.78%), whilst it rises to almost 5% in the other CD groups (4.68% in PCD and TCD and 4.23% in ACD). The comparisons between the relative abundances of taxa that yielded statistically significant differences are presented in Table 4. Within the phyla, *Proteobacteria* are increased in CD, especially in RCD, with respect to C. At the family level, again, the RCD condition differs from the other CD groups since it displays a marked reduction of *Fusobacteriaceae* and *Lachnospiraceace*. Noteworthily, within genera, *Neisseria* spp. presents a trend overlapping what was observed at the mucosal level, being critically increased in PCD, ACD, and RCD and close to C in TCD. 

### 3.4. Taxonomy-Based Analyses of Fecal Community

From Table 5, it emerges that TCD and RCD display values of relative abundances of phyla similar to C whereas ACD invariably shows a profile significantly different from TCD, except for *Proteobacteria*. At the family level, all CD groups are characterised by an increase of *Ruminococcaceae*, while *Veillonellaceae* appear decreased in ACD. Moreover, PCD displays an increased abundance in *Erysipelitrichaceae*, while RCD has an increase in *Pasteurellaceae*, which are suppressed in the other CD groups in comparison to C. Within genera, *Blautia*, *Coprococcus*, and *Roseburia* spp. show a strong increase in ACD in comparison to all the other groups, together with *Ruminococcus* spp. that appears increased even in PCD. Interestingly, *Veillonella* spp. and *Haemophilus* spp strongly increase in RCD while *Bifidobacterium* spp. and *Parabacteriodes* spp. abundance is negligible with respect to all the other groups. Finally, at the species level, the great increase of *Faecalibacterium prausnitzii* and *Veillonella dispar* emerges in RCD, whilst *Bifodobacterium longum*, that is increased in both PCD and ACD, appears suppressed. Also, *Roseburia faecis* appeares increased in ACD in comparison with other groups, at variance with *Bacteroides eggerthii* that displays negligible levels in both ACD and RCD, whilst it is increased in TCD.

**Figure 5:**
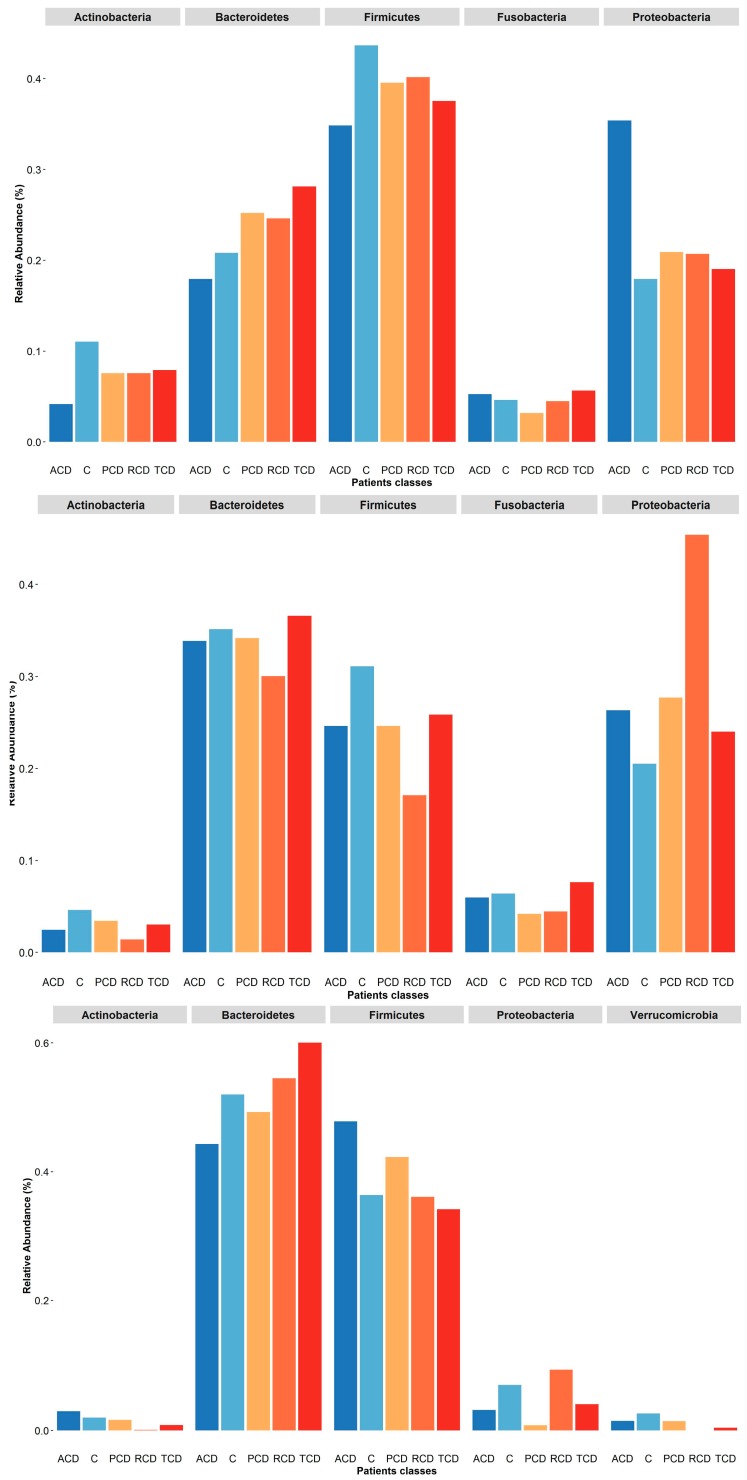
Plot of relative abundances of the five most abundant phyla retrieved in each sample biotype: At mucosal level (upper panel), although *Firmicutes* is the most abundant phylum, it is reduced in the ACD group as *Actinobacteria*. A parallel increase of the *Proteobacteria* is found in this group, while an expansion of *Bacteroidetes* in the RCD group and a reduction of *Fusobacteria* in the PCD one are evident. In the salivary samples (central panel), the most abundant phylum is *Bacteroidetes*. Again, *Firmicutes* is reduced in the ACD group as *Actinobacteria* in comparison to the C group. Interestingly, the RCD group shows a decrease of all phyla but *Proteobacteria*. In the stool samples (lower panel), the two most abundant phyla are *Bacteroidetes* and *Firmicutes*, with the other phyla showing negligible values. Noteworthily, the former is decreased in the ACD group and increased in the TCD group in comparison to C, while the latter is increased in the ACD and PCD groups.

**Figure 6:**
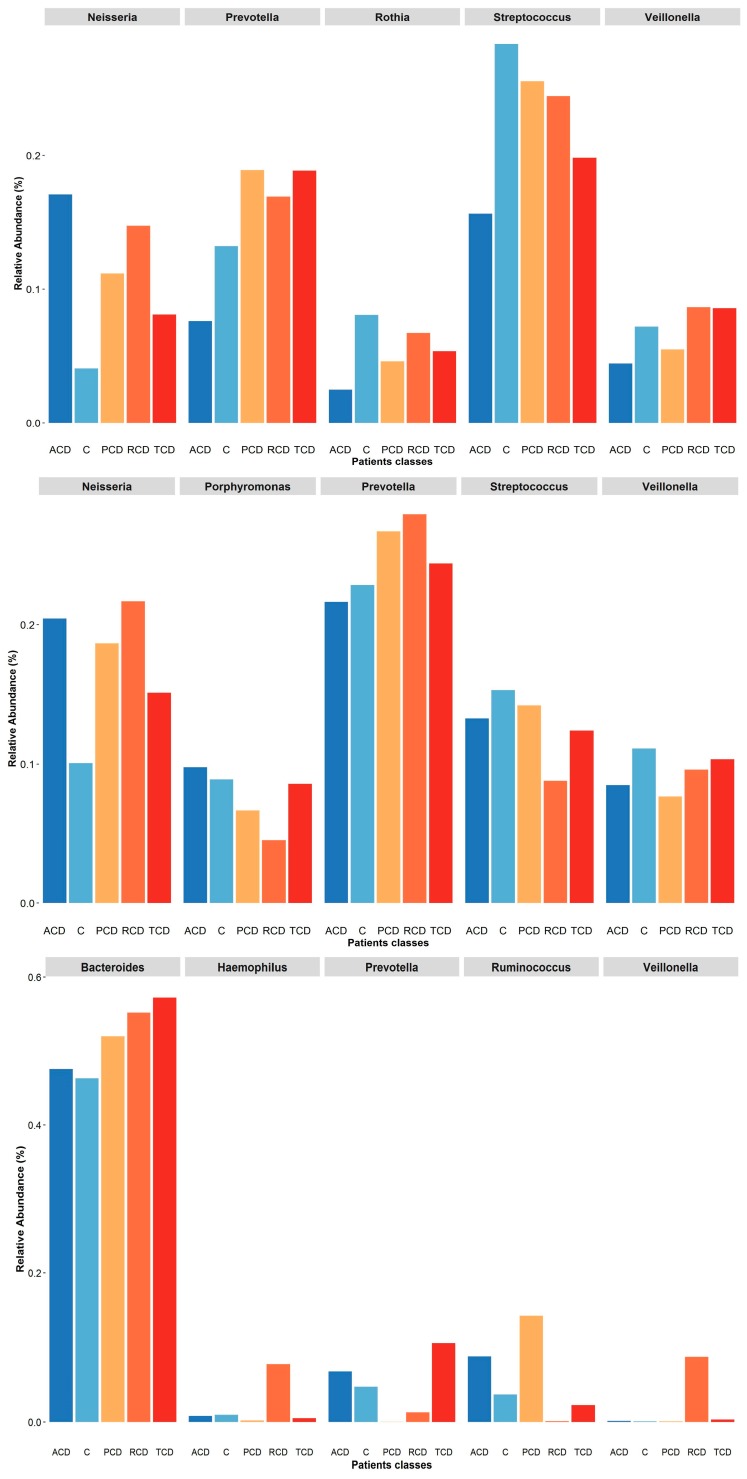
Plot of relative abundances of the five most abundant genera retrieved in each sample biotype: At the mucosal level (upper panel), all the most abundant genera are decreased in the ACD group but *Neisseria* as in all the other celiac groups with respect to the C one. Interestingly, *Prevotella* abundance is increased in the PCD, RCD, and TCD groups. In the salivary samples (central panel), again, *Neisseria* abundance is critically increased in all celiac groups with respect to the C one while *Prevotella* is increased only in the PCD, RCD, and TCD groups. Finally, in the stool samples (lower panel), the most abundant genus is *Bacteroides* that is increased in all celiac groups, with the other genera showing negligible values. Notably, the *Haemophilus* and *Veillonella* genera are critically increased only in the RCD group while the *Prevotella* is absent in the PCD group that displays a robust increase of the *Ruminococcus* as in the ACD group.

In summary, the relative distribution of the most abundant phyla and genera in the study cohort is represented in Figure 5 and Figure 6, respectively. It becomes evident that the salivary profiles mirror the condition at the mucosal level better than the fecal ones. The distribution of the five most abundant families is shown in Figure S1: Plot of relative abundances of the five most abundant families retrieved in each sample biotype, where, again, *Neisseriaceae* represents the most abundant one in both ACD and RCD mucosal and salivary samples. 

## 4. Discussion

In spite of the considerable understanding of the mechanisms leading to mucosal injury [19], the role of environmental factors in CD pathogenesis remains elusive and, consequently, the treatment is far from optimal. Perturbations of the gut microbiota (dysbiosis) and viral infections have been suggested to trigger the first hit of mucosal inflammation [20,21]. In this regard, a series of elegant experiments has provided some mechanistic insights showing that both gluten- and amylase-trypsin inhibitor-derived peptides generated by pathobiont species, such as *Pseudomonas aeriginosa*, are able to disrupt the epithelial barrier, to activate protease-activated receptors-2 signalling, and to recruit intraepithelial lymphocytes in sensitized mice with a susceptible genetic background [22,23]. Nonetheless, gut mucosa may also harbour gluten-degrading bacteria, such as *Rothia* spp. [24] and *Lactobacillus* spp. [25], with the potential to dampen the harmful effects of gluten peptides. When considering studies carried out in adulthood CD, the few performed on duodenal mucosa invariably found a decrease in the relative abundance of *Firmicutes* and an increase of *Proteobacteria*, together with changes of microbiota structure and composition [6,26,27]. However, the small sample size and the lack of a control group do not allow any firm conclusions to be drawn. On the other side, in the vast majority of studies carried out in paediatric CD, the analyses were carried out on stool samples [28]. Since this analysis may miss changes associated with duodenal inflammation or may find others not causally related to the disease process, we sought to investigate the structure and composition of gut microbiota directly at the duodenal level, the main site of tissue injury. We found marked alterations of the ecological indices in CD in terms of reduction of bacterial richness and diversity with respect to dyspeptic patients used as controls. Worthy of note, those suffering from PCD displayed mucosal indices similar to those with active enteropathy (ACD and RCD). This evidence reinforces the hypothesis that a significant shift of the microbiota composition anticipates the development of mucosal lesions [20]. When considering the data obtained in the groups with active enteropathy, we found the lowest values of α-diversity indices in the three bacterial communities of the RCD group. ACD also showed a reduced microbial richness that, together with a *Proteobacteria*-rich microbiota, has been repeatedly associated with chronic inflammatory conditions [29], including CD [6,26,27]. The TCD group shows intermediate values of both α- and β-diversity comprised between those of ACD and C ones, thus confirming that the GFD does not completely restore a healthy microbiota [6,27].

Moving on to the taxonomic analysis, our results definitely confirm the decrease of *Firmicutes* and increase of *Proteobacteria* in the duodenal mucosa of ACD patients, whereas *Bacteroidetes* displayed a mixed pattern, being decreased in ACD and increased in all the other CD groups, mostly in RCD. Remarkably, Wacklin and coworkers identified *Proteobacteria* as the most abundant phylum in biopsies of TCD patients who suffered from persistent abdominal symptoms [6,27]. It is known that a balanced gut microbiota is capable of inhibiting uncontrolled expansion of *Proteobacteria*, while a bloom of this phylum has been proposed as reflecting an unstable structure [28,29]. Noteworthily, the profiles of the phyla distribution in salivary samples almost completely mirror those found at the mucosal level, except for *Proteobacteria* that were found predominantly increased in RCD, whilst no substantial correspondence with the other consortia was found in fecal samples.

A further interesting point is the enrichment of the genus *Neisseria* (phylum *Proteobacteria*) and the corresponding family *Neisseriaceae* in duodenal biopsies of ACD patients, in agreement with previous evidence [26]. Our study enlarges the picture since an increased abundance of *Neisseria* spp. is already evident in PCD, reaches its maximum in ACD and RCD, and then lowers in TCD, although without reaching control levels. This evidence reinforces the hypothesis that it represents a disease-triggering factor instead of a pure consequence of intestinal damage. Moreover, the behaviour of the gluten-degradator species *Rothia* [30] in RCD mucosa should be pointed out since its abundance was similar to controls and significantly higher than in the other CD groups. The reason remains elusive, although it is conceivable that a long-lasting presence of undigested gluten peptides in the gut lumen may select those species with a high capability of degrading them. On the other hand, the depletion of this species in PCD might contribute to loss of gluten tolerance since an incomplete digestion generates oligopeptides with high immunogenicity [2]. 

Finally, in the salivary ecosystem, it emerges that the SR1 phylum rises from a rate of less than 1% in controls and refractory patients to about 5% in PCD, ACD, and TCD. This is a relatively recently described phylum [31], of which the abundance increases in the oral cavity of subjects with periodontal disease [32]. Another particular trend observed was the critical decrease of both *Lechnospiraceae* and *Fusobacteriaceae* in RCD, whereas they were increased in mucosal samples of noncomplicated CD patients. The taxonomic composition of the fecal community also suggests the existence of a pattern specific for RCD if considering the strong increase of *Pasteurellaceae* and, within this family, of the genus *Haemophilus* spp., in accordance with Cheng et al. [33]. Other taxa generally thought to be protective against inflammation, such as *Faecalibacterium prausnitzii* [34], display a boom in RCD. Therefore, despite the obvious limited number of RCD cases, the presence of particular changes to the microbiota composition in both saliva and fecal samples offers important opportunities for screening those cases transitioning to RCD. In addition, the recent finding of full recovery of duodenal architecture and clinical picture in a patient suffering from RCD following fecal microbiota transplantation for *Clostridium difficilie* infection strengthens the relevance of our data [35].

Certainly, our work has some limitations, including the limited sample size. However, it should be pointed out that only those patients who agreed to collect all biological samples were enrolled and that PCD and RCD are rare conditions. Furthermore, despite the oral cavity hardly representing the environment at the duodenal level, we found that saliva displays a microbiota structure and composition more similar to mucosal ones than feces. We also aknowledge that dyspepsia does not represent a real control condition; nonetheless a peculiar dysbiosis, largely dominated by the phylum *Proteobacteria*, genus *Neisseria*, clearly emerged in CD, even before the development of enteropathy. Moreover, the presence of an altered community structure not only in ACD but also in TCD clearly points to the need for additional non-dietary therapies. Since these findings are largely retrievable in the oral cavity, salivary analysis seems a useful tool to capture CD specific signatures. Taken together, our data pave the way for larger studies and support the utility of gut microbiota manipulation for preventive and therapeutic purposes in adulthood CD.

## Figures and Tables

**Table 1:** Variables in the celiac population.

Variable	With Villous Atrophy	Without Villous Atrophy
Gluten-containing diet	Active celiac disease	Potential celiac disease
Gluten-free diet	Refractory celiac disease	Treated celiac disease

**Table 2:** Demographic and clinical features of study cohort.

	Celiac Patients				Controls
	Potential	Active	Treated	Refractory	Functional Dyspepsia
Number of cases female:male ratio	6 3:3	13 11:2	29 20:9	4 4:0	31 24:7
Mean age at enrolment in years (± standard deviation)	41 ± 14	35 ± 6	37 ± 6	53 ± 15	44 ± 17
Median body mass index as kg/h^2^ (25th–75th)	22.4 (19.1–25.6)	21.3 (19.8–22.7)	20.5 (19.9–26.1)	16.9 (15.4–17.0)	22.1 (20.0–24.2)
Median time on a GFD in years (range)	5 cases no GFD 1 case on GFD since one year	No GFD	3.0 (2–7)	17 (11.2–22)	No GFD
Good adherence to GFD (score 3–4)	1	Not applicable	27	4	Not applicable
Poor adherence to GFD (score 0–2)	0	Not applicable	2	0	Not applicable
Autoimmunity	1	4	10	1	8
HLA-DQ2 ^+ve^	6	8	17	3	10
HLA-DQ8 ^+ve^	0	0	1	1	3
HLA-DQ2/DQ8 ^+ve^	0	3	2	0	1
HLA-DQ2/DQ8 ^−ve^	0	0	0	0	8
Unknown	0	2	10	0	9

Abbreviations: GFD: gluten-free diet; HLA: Human Leukocyte Antigen; ^+ve^: positive; ^−ve^: negative.

**Table 3:** Taxa displaying significantly different relative abundances in mucosal biopsies.

Taxon	Relative Abundance (%)					Comparisons * in Which Statistical Analysis Produced *p*-Values < 0.05
	C	PCD	TCD	ACD	RCD	
**Phyla**						
*Bacteroidetes*	20.76	25.19	28.08	18.20	24.60	C versus TCD (0.021) C versus TCD versus ACD (0.017) TCD versus ACD (0.019)
*Actinobacteria*	11.1	7.57	7.94	4.15	7.56	C versus TCD versus ACD (0.030) C versus ACD (0.012) C versus PCD versus ACD (0.035) TCD versus ACD (0.038)
*Proteobacteria*	17.89	20.92	19.21	35.48	20.71	C versus TCD versus ACD (0.045) C versus ACD (0.016) TCD versus ACD (0.036)
**Families**						
Phylum: *Firmicutes*						
*Streptococcaceae*	25.77	23.39	18.34	22.77	23.10	C versus TCD (0.041)
*Gemellaceae*	2.17	0.91	1.51	0.83	2.30	C versus TCD versus ACD (0.027) C versus ACD (0.011) C versus PCD versus ACD (0.022)
*Veillonellaceae*	7.37	6.54	8.95	4.50	9.39	C versus TCD versus ACD (0.035) T versus ACD (0.0090) ACD versus RCD (0.035)
*Lachnospiraceae*	2.71	2.50	3.26	2.00	1.67	TCD versus ACD (0.023)
Phylum: *Bacteroidetes*						
*Prevotellaceae*	12.1	17.95	17.80	6.80	15.93	C versus TCD versus ACD (0.0030) PCD versus ACD (0.045) TCD versus ACD (0.00040) ACD versus RCD (0.020)
Phylum: *Actinobacteria*						
*Micrococcaceae*	7.51	4.44	4.98	2.27	6.26	C versus ACD (0.029) ACD versus RCD (0.0061)
Phylum: *Proteobacteria*						
*Neisseriaceae*	3.95	10.46	7.91	16.14	14.90	C versus ACD (0.034)
**Genera**						
Phylum: *Firmicutes*; Family: *Streptococcaceae*						
*Streptococcus* spp.	28.17	25.52	19.76	15.60	24.42	C versus TCD (0.023) C versus TCD versus ACD (0.013) C versus PCD versus ACD (0.048) C versus ACD (0.016)
Phylum: *Firmicutes*; Family: *Peptoniphilaceae*						
*Parvimonas* spp.	0.74	1.39	0.45	0.32	0.34	PCD versus ACD (0.020) C versus PCD versus ACD (0.038)
Phylum: *Firmicutes*; Family: *Veillonellaceae*						
*Veillonella* spp.	7.2	5.49	8.57	4.44	8.65	C versus TCD versus ACD (0.038) TCD versus ACD (0.009)
Phylum: *Proteobacteria*; Family: *Neisseriaceae*						
*Neisseria* spp.	4.07	11.16	8.10	17.02	14.74	C versus PCD versus ACD (0.038) C versus ACD (0.034) C versus TCD versus ACD (0.049)
Phylum: *Bacteroidetes*; Family: *Prevotellaceae*						
*Prevotella* spp.	13.18	18.90	18.82	7.55	16.92	ACD versus RCD (0.020) PCD versus ACD (0.026) TCD versus ACD (0.0004)
Phylum: *Actinobacteria*; Family: *Actinomycetaceae*						
*Actinomyces* spp.	1.66	1.71	1.42	0.66	0.82	TCD versus ACD (0.044)
Phylum: *Actinobacteria*; Family: *Micrococcaceae*						
*Rothia* spp.	8.10	4.60	5.35	2.47	6.72	ACD versus RCD (0.0061) C versus ACD (0.027)
**Species**						
Phylum: *Bacteroidetes*; Family: *Prevotellaceae*						
*Prevotella melaninogenica*	23.1	35.71	32.5	14.36	29.67	C versus TCD (0.035) C versus TCD versus ACD (0.0052) PCD versus ACD (0.015) TCD versus ACD (0.0019)
*Prevotella copri*	0.73	1.32	1.07	1.59	2.58	TCD versus ACD (0.027)
*Prevotella pallens*	3.24	2.84	4.85	1.40	1.58	TCD versus ACD (0.034)
*Prevotella nigrescens*	0.26	2.45	0.46	0.38	1.42	C versus PCD versus ACD (0.003) PCDversus ACD (0.003) C versus PCD (0.002)
Phylum: *Actinobacteria*; Family: *Micrococcaceae*						
*Rothia aerea*	0.45	0.080	0.47	0.040	1.13	RCD versus TCD (0.026) TCD versus ACD (0.025) C versus ACD (0.034) ACD versus RCD (0.0009)
*Rothia mucilaginosa*	19.17	12.47	13.13	10.00	17.74	ACD versus RCD (0.045)

Abbreviations: C = controls; PCD = potential celiac disease, ACD = active celiac disease; TCD = treated celiac disease; RCD = refractory celiac disease. * The following comparisons were performed: C versus PCD; C versus TCD; C versus ACD; C versus TCD versus ACD; C versus ACD; PCD versus ACD; PCD versus ACD versus C; TCD versus RCD; ACD versus TCD; and ACD versus RCD.

**Table 4:** Taxa displaying significantly different relative abundances in saliva samples.

Taxon	Relative Abundance (%)					Comparisons * in Which Statistical Analysis Produced *p*-Values < 0.05
	C	PCD	TCD	ACD	RCD	
**Phyla**						
*Proteobacteria*	20.51	27.71	24.03	26.35	45.11	TCD versus RCD (0.024)
**Families**						
Phylum: *Fusobacteria*						
*Fusobacteriaceae*	4.50	2.95	5.43	4.16	1.66	ACD versus RCD (0.025) TCD versus RCD (0.0088)
Phylum: *Firmicutes*						
*Lachnospiraceae*	1.11	0.66	0.97	0.82	0.22	TCD versus RCD (0.0038) ACD versus RCD (0.033)
**Genera**						
Phylum: *Proteobacteria*; Family: *Neisseriaceae*						
*Neisseria* spp.	10.08	18.66	15.10	20.44	21.69	C versus ACD (0.049)
Phylum: *Proteobacteria*; Family: *Moraxellaceae*						
*Acinetobacter* spp	2.08	0.0099	0.068	0.016	0.069	C versus ACD (0.032)
Phylum: *Fusobacteria*; Family: *Fusobacteriaceae*						
*Fusobacterium* spp.	4.62	2.99	5.54	4.27	2.44	TCD versus RCD (0.032)
Phylum: *Firmicutes*; Family: *Lachnospiraceae*						
*Oribacterium* spp.	0.40	0.21	0.38	0.26	0.09	TCD versus ACD (0.038) TCD versus RCD (0.037)
**Species**						
Phylum: *Bacteroidetes*; Family: *Porphyromonadaceae*						
*Porphyromonas endodontalis*	1.35	0.65	0.76	4.07	1.66	C versus T versus A (0.039)

**Table 5:** Taxa displaying significantly different relative abundances in stool samples.

Taxon	Relative Abundance (%)					Comparisons * in Which Statistical Analysis Produced *p* Values < 0.05
	C	PCD	TCD	ACD	RCD	
**Phyla**						
*Bacteroidetes*	51.97	49.23	59.99	44.27	54.5	TCD versus ACD (0.0027) C versus TCD versus ACD (0.025)
*Firmicutes*	36.39	42.3	34.21	47.83	36.10	TCD versus ACD (0.020)
*Actinobacteria*	1.96	4.6	0.82	2.93	0.098	TCD versus ACD (0.029)
*Proteobacteria*	6.90	0.78	3.96	3.12	9.27	ACD versus RCD (0.044)
*Coriobacteriaceae*	0.14	0.67	0.12	1.39	0.0093	C versus TCD versus ACD (0.0066) C versus PCD versus ACD (0.015) TCD versus ACD (0.0063) ACD versus RCD (0.044)
**Families**						
Philum: *Firmicutes*						
*Clostridiaceae*	0.18	0.23	0.57	0.63	0.11	C versus TCD versus ACD (0.020) C versus PCD versus ACD (0.029) C versus TCD (0.037)
*Veillonellaceae*	6.35	4.66	6.35	2.40	8.85	C versus ACD (0.046)
*Erysipelitrichaceae*	0.30	2.21	0.44	1.14	0.12	C versus TCD versus ACD (0.0090) C versus PCD versus ACD (0.012) TCD versus ACD (0.0070) C versus ACD (0.0050)
*Ruminococcaceae*	23.52	23.81	13.94	23.52	18.47	TCD versus ACD (0.016)
Philum: *Actinobacteria*						
*Coriobacteriaceae*	0.14	0.67	0.12	1.39	0.009	C versus PCD versus ACD (0.015)
Philum: *Proteobacteria*						
*Enterobacteriaceae*	0.46	0.27	2.13	1.84	0.01	TCD versus RCD (0.038) ACD versus RCD (0.028)
*Pasteurellaceae*	2.32	0.17	0.41	0.56	5.86	TCD versus RCD (0.028) ACD versus RCD (0.044)
**Genera**						
Philum: *Firmicutes*; Family: *Lachnospiraceae*						
*Blautia* spp.	0.53	0.94	0.88	3.12	0.46	C versus TCD versus ACD (0.016) C versus ACD (0.0059) C versus PCD versus ACD (0.022)
*Coprococcus* spp.	0.31	0.57	0.47	1.10	0.094	C versus TCD versus ACD (0.008) C versus ACD (0.0030) C versus PCD versus ACD (0.011) TCD versus ACD (0.017)
*Roseburia* spp.	0.81	0.17	1.50	1.72	0.39	PCD versus ACD (0.043)
Philum: *Firmicutes*; Family: *Ruminococcaceae*						
*Ruminococcus* spp.	3.62	14.22	2.22	8.75	0.12	ACD versus RCD (0.044) TCD versus ACD (0.047) PCD versus ACD versus C (0.037) TCD versus RCD (0.030)
Philum: *Firmicutes*; Family: *Clostridiaceae*						
*Dorea* spp.	0.88	0.36	0.3	1.03	0.19	C versus TCD versus ACD (0.008) C versus ACD (0.012) C versus PCD versus ACD (0.027) TCD versus ACD (0.0031)
Philum: *Firmicutes*; Family: *Veillonellaceae*						
*Veillonella* spp.	0.095	0.021	0.32	0.16	8.67	RCD versus TCD (0.029) ACD versus RCD (0.028)
Philum: *Firmicutes*; Family: *Acidaminococcaceae*						
*Megasphaera* spp.	0.079	0.0037	1.20	0.02	0.08	RCD versus TCD (0.044)
Philum: *Proteobacteria*; Family: *Pasteurellaceae*						
*Haemophilus* spp.	0.96	0.19	0.50	0.80	7.69	TCD versus RCD (0.038) ACD versus RCD (0.043)
Philum: *Actinobacteria*; Family: *Bifidobacteriaceae*						
*Bifidobacterium* spp.	1.65	1.15	0.89	2.62	0.11	TCD versus ACD (0.045)
Philum: *Bacteroidetes*; Family: *Porphyromonadaceae*						
*Parabacteriodes* spp.	6.89	2.77	4.23	3.11	1.41	TCD versus ACD (0.029)
**Species**						
Philum: *Firmicutes*; Family: *Lachnospiraceae*						
*Roseburia fecis*	0.99	0.19	0.93	2.76	0.74	C versus TCD versus ACD (0.049) C versus PCD versus ACD (0.044) C versus ACD (0.022)
Philum: *Firmicutes*; Family: *Ruminococcaceae*						
*Faecalibacterium prausnitzii*	13.90	11.05	9.49	15.42	27.48	TCD versus RCD (0.049)
Philum: *Firmicutes*; Family: Veillonellaceae						
*Veillonella dispar*	0.45	0.15	1.77	0.80	22.44	TCD versus RCD (0.029) ACD versus RCD (0.027)
Philum: *Actinobacteria*; Family: *Bifidobacteriaceae*						
*Bifodobacterium longum*	1.40	2.07	0.80	4.99	0.008	C versus TCD versus ACD (0.011) C versus ACD (0.016) TCD versus ACD (0.004) C versus PCD versus ACD (0.045)
Philum: *Bacteroidetes*; Family: *Bacteroidaceae*						
*Bacteroides eggerthii*	2.49	11.07	6.68	0.46	0	TCD versus ACD (0.046)

## Supplementary Materials

The following are available online at https://www.mdpi.com/2077-0383/9/4/1109/s1, Figure S1: Plot of relative abundances of the five most abundant families retrieved in each sample biotype; Table S1: Taxonomic assignment across sample biotypes (mucosal, salivary and fecal) in the study cohort; Table S2: α-diversity indices for each sample.

## Abbreviations

ACD	active celiac disease
C	control
CD	celiac disease
GFD	gluten-free diet
OTU	operational taxonomic unit
PCD	potential celiac disease
RCD	refractory celiac disease
spp	species
TCD	treated celiac disease
