# Supplementary material for: Comparative Study of Salivary, Duodenal, and Fecal Microbiota Composition Across Adult Celiac Disease
Source: J Clin Med. 2020 Apr 13;9(4):1109. doi: 10.3390/jcm9041109 (PMC7231226; doi:10.3390/jcm9041109)
Supplement: Supplementary file 1 [file jcm-09-01109-s001.zip › jcm-747128 supplementary/jcm-747128 Figure S1,Table S1 .docx]

**Supplemental Figure S1. Plot of relative abundances of the five most abundant families retrieved in each sample biotype.**

At mucosal level (panel A), the *Streptococcaceae* is the most abundant family in all study groups, although it appears reduced in celiac ones. Interestingly, active celiac patients display a reduced abundancy of all families, except for *Neisseriaceae* that is tremendously increased even in the other forms of the disease. Moreover, a parallel increase of both *Prevotellaceae* and *Veillonellaceae* is found in all celiac groups, but active one. In the salivary samples (panel B), the most abundant family is *Prevotellaceae* that is increased in the potential form of the disease. Notably, the refractory group shows a decrease of *Porphyromonadaceae*, *Streptococcaceae* and *Veillonellaceae* in comparison to the control group. Again, *Neisseriaceae* is increased in all celiac groups with respect to the control one. In the stool samples (panel C), the two most abundant phyla are *Bacteroidaceae* and *Ruminococcaceae*. Noteworthy, the former is decreased in the active celiac group and increased in the other celiac groups in comparison to control group, while the latter is increased in all celiac groups, except in treated one. Moreover, the *Lechnospiraceae* is also increased in active celiacs, while the *Prevotellaceae* is absent in potential celiacs and increased in treated ones. Abbreviations for patient cohorts: A: active celiac disease; C: controls; P: potential celiac disease; R: refractory celiac disease, T: treated celiac disease.


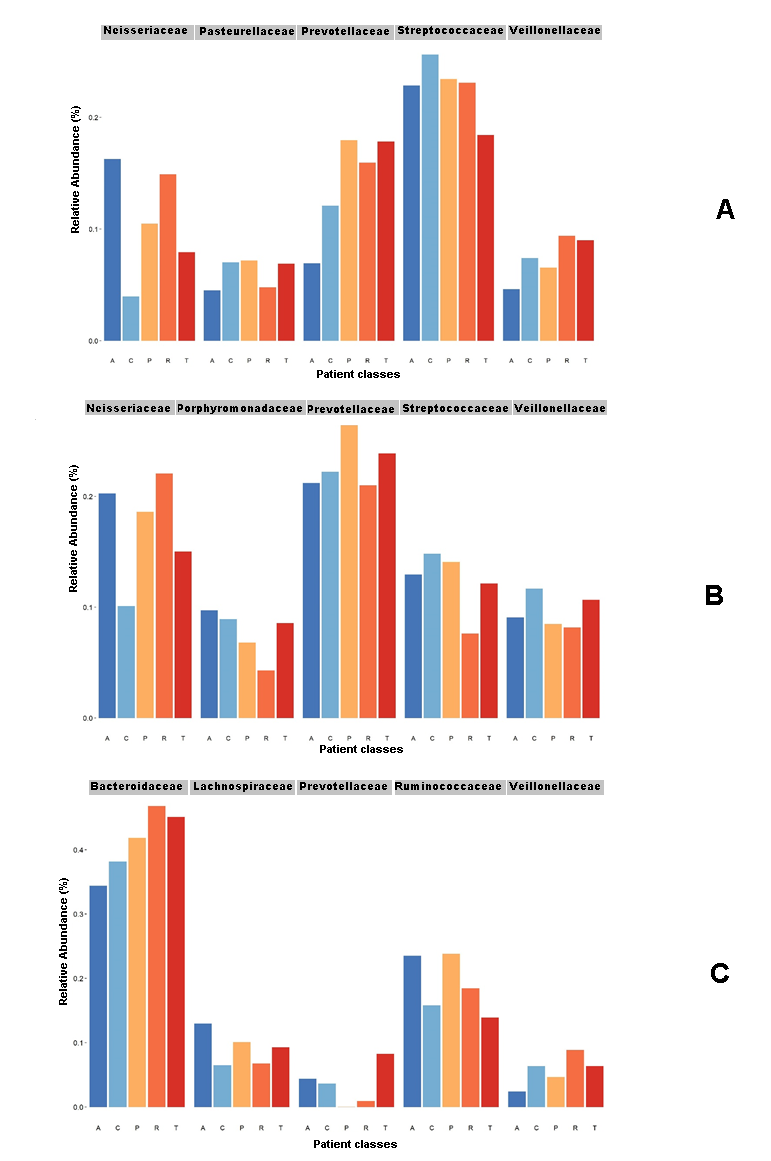


**Supplemental Table** **S1**. **Taxonomic assignment across sample biotypes (mucosal, salivary and fecal) in the study cohort.**

| **Taxonomic Assignment** | | | |
| --- | --- | --- | --- |
| **Number of assigned**  **OTU per biotype** | **Mucosal** | **Salivary** | **Fecal** |
|  | 2589 | 1593 | 4245 |
|  | - 24 phyla - 48 classes - 82 orders - 147 families - 273 genera - 160 species | - 19 phyla - 36 classes - 62 orders - 109 families - 167 genera - 91 species | - 19 phyla - 34 classes - 55 orders - 103 families - 180 genera - 113 species |

Abbreviation. OTU: Operational Taxonomic Unit.
