# Supplementary material for: Comparative Study of Salivary, Duodenal, and Fecal Microbiota Composition Across Adult Celiac Disease
Source: J Clin Med. 2020 Apr 13;9(4):1109. doi: 10.3390/jcm9041109 (PMC7231226; doi:10.3390/jcm9041109)
Supplement: Supplementary file 1 [file jcm-09-01109-s001.zip › jcm-747128 supplementary/Supplementary Table 2.pdf]

Supplemental Table 2. α-diversity indices for each sample

SampleID	Sample	Group	Observed Richness	Chao1	Shannon
42565F3956	mucosa	A	84	101	3,32
42567F3956	mucosa	A	74	112,5	0,65
66257F6172	mucosa	A	95	182,35	3,2
66258F6172	mucosa	A	15	106	2,64
66261F6173	mucosa	A	344	432,02	3,69
66264F6173	mucosa	A	118	278,91	3,88
66268F6173	mucosa	A	66	105	1,56
66270F6174	mucosa	A	28	97	3,27
92072F7971	mucosa	A	338	494,42	4,65
92074F7971	mucosa	A	306	415,45	3,76
92082F7972	mucosa	A	272	451,15	3,2
92088F7973	mucosa	A	240	378,49	4,53
136251F1290	mucosa	C	271	410,47	1,9
136252F1290	mucosa	C	334	455,23	4,31
136253F1290	mucosa	C	430	585,82	4,43
136254F1290	mucosa	C	352	482,78	3,58
136255F1290	mucosa	C	305	410	3,8
159064F1530	mucosa	C	87	168,67	4,17
159065F1530	mucosa	C	86	142	4,02
159066F1530	mucosa	C	341	470,49	4
159067F1530	mucosa	C	373	533,53	3,29
159068F1530	mucosa	C	181	269,77	3,51
159069F1530	mucosa	C	371	433,35	4,06
42568F3956	mucosa	C	212	257,32	3,84
42570F3957	mucosa	C	254	311,4	4,18
42573F3957	mucosa	C	260	333,2	3,51
42579F3957	mucosa	C	236	293,95	4,26
42581F3958	mucosa	C	318	353,36	4,14
42583F3958	mucosa	C	204	238,44	3,98
66266F6173	mucosa	C	12	27	2,44
92065F7970	mucosa	C	127	289	4,3
92067F7971	mucosa	C	372	502,02	3,57
92075F7971	mucosa	C	294	354,67	4,58
92076F7971	mucosa	C	233	409,13	3,82
92077F7972	mucosa	C	192	313,36	4,22
92081F7972	mucosa	C	141	218,9	4
92084F7972	mucosa	C	248	522,68	4,19
92086F7972	mucosa	C	494	740,5	4,94
92087F7973	mucosa	C	292	456,92	4,61
136261F1290	mucosa	P	294	415,73	4
42582F3958	mucosa	P	333	398,81	4,51
66259F6173	mucosa	P	370	451,33	3,9
92069F7971	mucosa	P	354	468,49	4,61

92070F7971	mucosa	P	350	518,49	3,83
159071F1530	mucosa	R	370	475,39	3,95
42566F3956	mucosa	R	198	228	3,19
42572F3957	mucosa	R	222	277	3,84
92073F7971	mucosa	R	296	409,14	3,32
92078F7972	mucosa	R	307	453,39	4,18
136256F1290	mucosa	T	159	252,72	4,68
136257F1290	mucosa	T	286	406,66	4,05
136258F1290	mucosa	T	447	588,08	4,18
136259F1290	mucosa	T	417	584,19	4,98
136260F1290	mucosa	T	406	553,42	4,51
159070F1530	mucosa	T	401	488,55	3,76
42569F3956	mucosa	T	265	314,04	3,7
42571F3957	mucosa	T	141	156,81	3,82
42574F3957	mucosa	T	269	345,96	3,55
42576F3957	mucosa	T	335	396,57	3,86
42578F3957	mucosa	T	107	122,55	3,93
42580F3958	mucosa	T	228	300,06	3,98
42584F3958	mucosa	T	321	405	3,7
66260F6173	mucosa	T	320	393,24	3,55
66262F6173	mucosa	T	304	420,73	3,56
66263F6173	mucosa	T	282	369,26	2,91
66265F6173	mucosa	T	26	83,75	3,16
66267F6173	mucosa	T	56	126	3,67
66269F6174	mucosa	T	169	248,8	3,72
66296F6176	mucosa	T	344	462,7	3,97
92064F7970	mucosa	T	253	333,1	4,1
92066F7970	mucosa	T	383	558,66	4,31
92068F7971	mucosa	T	381	520,09	3,98
92071F7971	mucosa	T	255	342,02	3,6
92079F7972	mucosa	T	395	617,82	4,43
92080F7972	mucosa	T	296	464,81	3,88
92085F7972	mucosa	T	303	429,04	4,86
92089F7973	mucosa	T	254	380,67	4,27
42534F3952	stool	A	302	450,17	3,12
42536F3952	stool	A	555	822,5	3,96
66271F6174	stool	A	910	1204,05	4,44
66273F6174	stool	A	787	1109,88	4,82
66276F6174	stool	A	1275	1576,34	5,31
66278F6174	stool	A	982	1425,31	4,2
66284F6175	stool	A	912	1222,04	4,83
66286F6175	stool	A	1118	1483,45	4,96
66288F6175	stool	A	823	1163,18	4,53
66290F6176	stool	A	500	776,39	3,97
92031F7967	stool	A	1175	1526,15	4,43
92032F7967	stool	A	576	890,53	3,63
136262F1290	stool	C	843	1232,89	4,51
136263F1290	stool	C	634	860,14	4,07
136264F1290	stool	C	682	1024,35	3,84
159053F1530	stool	C	693	953,44	4,06

159054F1530	stool	C	713	1028,16	4,39
159055F1530	stool	C	1036	1303,52	4,57
42537F3952	stool	C	173	273,04	3,98
42539F3953	stool	C	358	580,47	3,29
42545F3954	stool	C	409	617,5	3,83
66281F6175	stool	C	874	1129,01	3,97
66283F6175	stool	C	415	641,62	0,5
66292F6176	stool	C	66	91,67	2,57
66293F6176	stool	C	722	1059,25	3,98
92026F7966	stool	C	622	970,43	3,03
92027F7967	stool	C	856	1206,24	4,28
92033F7967	stool	C	734	1099,05	4,4
92036F7967	stool	C	307	514,24	3,36
92038F7968	stool	C	688	1065,48	3,07
66275F6174	stool	P	1080	1439,03	4,05
66294F6176	stool	P	615	947,28	4,21
92029F7967	stool	P	571	825,43	3,68
42535F3952	stool	R	503	666,38	4,11
42540F3953	stool	R	711	930,03	3,56
136265F1290	stool	T	932	1291,78	4,6
42538F3953	stool	T	508	703,64	3,98
42541F3953	stool	T	290	416,48	3,33
42542F3953	stool	T	287	452,51	4,44
42544F3954	stool	T	469	718,56	3,95
66272F6174	stool	T	859	1192,29	4,19
66274F6174	stool	T	612	942,37	4,5
66277F6174	stool	T	1007	1348,26	4,15
66279F6175	stool	T	1105	1456,75	4,77
66280F6175	stool	T	755	1015,16	4,4
66282F6175	stool	T	824	1235,21	4,48
66285F6175	stool	T	853	1162,4	4,65
66287F6175	stool	T	1060	1346,35	4,75
66289F6176	stool	T	707	1027,91	4,54
66291F6176	stool	T	705	985,84	4,14
66295F6176	stool	T	710	1018,9	4,36
92025F7966	stool	T	652	1007,44	3,89
92028F7967	stool	T	739	1043,17	4,32
92030F7967	stool	T	606	847,68	3,65
92034F7967	stool	T	816	1141,25	3,93
92037F7968	stool	T	1161	1551,28	4,8
42546F3954	saliva	A	294	360,28	2,72
42548F3954	saliva	A	379	417,52	3,77
66239F6171	saliva	A	414	505,02	3,17
66241F6171	saliva	A	503	690,2	3,35
66245F6171	saliva	A	401	482,43	3,4
66249F6172	saliva	A	248	287,33	3,02
66252F6172	saliva	A	265	356,5	3,54
66254F6172	saliva	A	391	490,02	3,75
66256F6172	saliva	A	269	361,12	3,22
92046F7968	saliva	A	391	521,8	3,36

92047F7969	saliva	A	540	651,02	4,2
92048F7969	saliva	A	302	389,26	3,54
92061F7970	saliva	A	406	519,75	4,06
92063F7970	saliva	A	445	558,56	4,12
136266F1290	saliva	C	138	166,33	2,86
136267F1290	saliva	C	305	444,44	3,46
136268F1290	saliva	C	371	530	3,31
136269F1290	saliva	C	307	394,6	3,51
159058F1530	saliva	C	296	417,54	3,77
159059F1530	saliva	C	397	529,26	4,03
159060F1530	saliva	C	417	546,5	4,15
159061F1530	saliva	C	442	559,86	3,72
42549F3954	saliva	C	297	358,39	3,65
42551F3955	saliva	C	398	456,18	3,94
42554F3955	saliva	C	213	237,41	2,91
42560F3955	saliva	C	400	491,24	4,1
42562F3956	saliva	C	416	501,13	3,81
42564F3956	saliva	C	429	482,2	3,63
66251F6172	saliva	C	352	459,06	2,78
92040F7968	saliva	C	337	428,74	3,94
92042F7968	saliva	C	384	465,16	3,51
92049F7969	saliva	C	406	589,86	3,36
92050F7969	saliva	C	282	383,79	3,44
92051F7969	saliva	C	439	602,88	3,88
92055F7969	saliva	C	292	385,37	3,88
92057F7970	saliva	C	313	388	3,47
92059F7970	saliva	C	426	530,07	3,84
92060F7970	saliva	C	330	421,74	3,88
136275F1290	saliva	P	400	513,17	3,29
42563F3956	saliva	P	444	559,62	3,79
66243F6171	saliva	P	357	432,35	3,64
92044F7968	saliva	P	312	423,89	3,56
159063F1530	saliva	R	342	445,64	2,54
42547F3954	saliva	R	198	240	2,57
42553F3955	saliva	R	250	309,29	3,37
92052F7969	saliva	R	273	344	3,84
136270F1290	saliva	T	284	370,06	3,2
136271F1290	saliva	T	374	438,77	3,8
136272F1290	saliva	T	424	507,08	4,1
136273F1290	saliva	T	415	510,07	3,87
136274F1290	saliva	T	449	599,8	3,84
159062F1530	saliva	T	341	462,87	3,72
42550F3954	saliva	T	414	493,23	3,27
42552F3955	saliva	T	262	315,1	2,55
42555F3955	saliva	T	277	337,38	3,24
42557F3955	saliva	T	414	466,13	3,58
42559F3955	saliva	T	297	382,04	3,87
42561F3956	saliva	T	342	477,03	3,32
42588F3956	saliva	T	431	508,34	3,08
66240F6171	saliva	T	394	521,57	4,01

66242F6171	saliva	T	324	416,94	3,73
66244F6171	saliva	T	339	481,6	3,24
66246F6171	saliva	T	359	452,95	4,03
66247F6171	saliva	T	341	418	3,75
66248F6171	saliva	T	245	322,66	2,7
66250F6172	saliva	T	392	505,17	3,91
66253F6172	saliva	T	353	423,29	3,59
66255F6172	saliva	T	371	480,44	3,76
92039F7968	saliva	T	411	525,47	3,55
92041F7968	saliva	T	339	457,83	4,1
92043F7968	saliva	T	441	581,93	3,86
92045F7968	saliva	T	283	406,1	3,15
92053F7969	saliva	T	377	477,09	4,26
92054F7969	saliva	T	320	383,07	3,3
92058F7970	saliva	T	435	519,06	3,9
92062F7970	saliva	T	481	592,2	4,31

A: active coeliac disease; C: controls; P: potential coelic disease; R: refractory coeliac disease; T: treated coeliac disease.
